# Mechanistic understanding of MeHg-Se antagonism in soil-rice systems: the key role of antagonism in soil

**DOI:** 10.1038/srep19477

**Published:** 2016-01-18

**Authors:** Yongjie Wang, Fei Dang, R. Douglas Evans, Huan Zhong, Jiating Zhao, Dongmei Zhou

**Affiliations:** 1 State Key Laboratory of Pollution Control and Resources Reuse, School of the Environment, Nanjing University, Nanjing 210023, P.R. China; 2 Key Laboratory of Soil Environment and Pollution Remediation, Institute of Soil Science, Chinese Academy of Sciences, Nanjing 210008, P.R. China; 3 Environmental and Resource Studies Program (ERS), Trent University, Peterborough, Ontario, Canada; 4 Environmental and Life Sciences Program (EnLS), Trent University, Peterborough, Ontario, Canada; 5 Key Lab for Biomedical Effects of Nanomaterial and Nanosafety, Laboratory of Metallomics and Metalloproteomics, Institute of High Energy Physics, Chinese Academy of Sciences, Beijing 100049, P.R. China

## Abstract

Methylmercury (MeHg) accumulation in rice has great implications for human health. Here, effects of selenium (Se) on MeHg availability to rice are explored by growing rice under soil or foliar fertilization with Se. Results indicate that soil amendment with Se could reduce MeHg levels in soil and grain (maximally 73%). In contrast, foliar fertilization with Se enhanced plant Se levels (3–12 folds) without affecting grain MeHg concentrations. This evidence, along with the distinct distribution of MeHg and Se within the plant, demonstrate for the first time that Se-induced reduction in soil MeHg levels (i.e., MeHg-Se antagonism in soil) rather than MeHg-Se interactions within the plant might be the key process triggering the decreased grain MeHg levels under Se amendment. The reduction in soil MeHg concentrations could be mainly attributed to the formation of Hg-Se complexes (detected by TEM-EDX and XANES) and thus reduced microbial MeHg production. Moreover, selenite and selenate were equally effective in reducing soil MeHg concentrations, possibly because of rapid changes in Se speciation. The dominant role of Se-induced reduction in soil MeHg levels, which has been largely underestimated previously, together with the possible mechanisms advance our mechanistic understanding about MeHg dynamics in soil-rice systems.

Recently, concerns about methylmercury (MeHg) accumulation in rice grain have been raised, mainly because consumption of mercury contaminated rice (up to 145 μg MeHg kg^–1^)^1^, in addition to fish, is considered to be an important pathway of human exposure to MeHg^2^. Accordingly, there is increasing interest in mercury-selenium (Hg-Se) antagonism in soil-rice systems, given that Se is known to protect mammals and aquatic organisms from mercury bioaccumulation and toxicity^3^,^4^,^5^,^6^,^7^,^8^. A growing body of work provides evidence that Se supplementation could reduce bioaccumulation and toxicity of inorganic mercury (IHg) to plants. For instance, IHg uptake by radish plants^9^ and garlic^10^ decreased under selenite or selenate addition and recent hydroponic studies have proposed that the inert IHg-Se complexes and/or high molecular weight proteinaceous complexes in the root under Se addition could be responsible for the observed antagonism between Se and IHg in plants^11^,^12^,^13^,^14^.

Much of the focus in the past decade has been on elucidating the IHg-Se interaction and thus Se-induced reduction in IHg bioaccumulation and toxicity; however, the effect of Se on MeHg bioaccumulation is less understood. This is unfortunate given that MeHg (highly toxic and bioaccumulative^15^) not IHg is the major concern in rice (*Oryza sativa* L.) when considering food safety. In fact, little information has been available about the potential effects of Se on MeHg accumulation in plants until recently when a pilot field survey demonstrated a downward trend in brown rice MeHg levels with increasing soil Se levels in field-collected samples from a mining-contaminated area^16^. Similar inhibition was also demonstrated in a recent pot experiment^17^. Suppression of MeHg translocation within plant and/or possible complexation between IHg and Se in soil were hypothesized to account for the reduced MeHg accumulation in rice following Se addition, i.e., MeHg-Se antagonism^16^,^17^.

These data are important and valuable in the evaluation of MeHg-Se antagonism. However, such studies are still scarce, with limited data on not only the relative importance of MeHg-Se antagonism in soil or plant, but also the underlying influencing factors. Recently, the inhibitory effect of Se addition on MeHg production by sulfate-reducing bacteria (SRB) was emphasized in bacterial culture experiments^18^,^19^. Therefore, it is reasonable to assume there would be a negative effect of added Se on MeHg production in soils (namely ‘antagonism in soil’) and thus MeHg bioaccumulation, considering that mercury methylation in soil/sediment by anaerobic bacteria is a key first step in determining concentrations and bioaccumulation of MeHg^20^. Unfortunately, in field-collected samples from mercury mining areas, the potential negative effect of Se on soil MeHg levels may be masked. Indeed, co-existence of Hg and Se in mercury mining areas^21^ resulted in positive relationships between MeHg and Se in soils^16^, which may obscure the MeHg-Se antagonism in soils. Therefore, the existence of MeHg-Se antagonism in soils, as well as its potential impact on soil MeHg levels and MeHg accumulation in rice warrants further investigation, as does the relative contribution of antagonism in soil to the process of MeHg accumulation in rice grain, compared to possible MeHg-Se interactions within plant (namely ‘antagonism within plant’).

Here we aim at addressing two fundamental questions regarding reduced MeHg accumulation in rice under Se amendment (i.e., MeHg-Se antagonism): (1) the main reason(s) for the MeHg-Se antagonism, i.e., reduced soil MeHg levels due to Se amendment or MeHg-Se interactions within plants and (2) factors (e.g., Se speciation, amended Se doses, ambient Se levels and Se fertilization approaches) controlling the MeHg-Se antagonism. Foliar fertilization with Se has been shown to effectively enhance tissue Se levels^22^,^23^ and our recent evidence suggests that the inhibitory effects of Se on Hg bioavailability depends on the chemical speciation of Se^24^. Therefore, the effect of both selenite and selenate (the main Se species for plant uptake from soil)^25^, in varying concentrations, as well as soil versus foliar fertilization with Se, on MeHg bioaccumulation will be explored using two soils with contrasting ambient Se levels (i.e., Low-Se and High-Se soils).

## Results

### Concentrations of Se and MeHg in Se-amended soils

Low-Se and High-Se soils differed distinctly in ambient Se concentrations (0.91 ± 0.10 and 10.55 ± 0.17 mg Se kg^–1^, respectively, Table 1). The resulting soil Se concentrations following soil fertilization were 1.4–7.7 fold and 1.0–1.3 fold greater than the control for Low-Se and High-Se soils, respectively (day 0, supplementary information (SI) Fig. S1). For both soils, soil Se concentrations did not vary significantly at the start and end of the experiments (two-tailed paired t-tests, *p* > 0.05), suggesting minor effects of Se uptake by plants or Se volatilization on soil Se levels.

**Figure 1:**
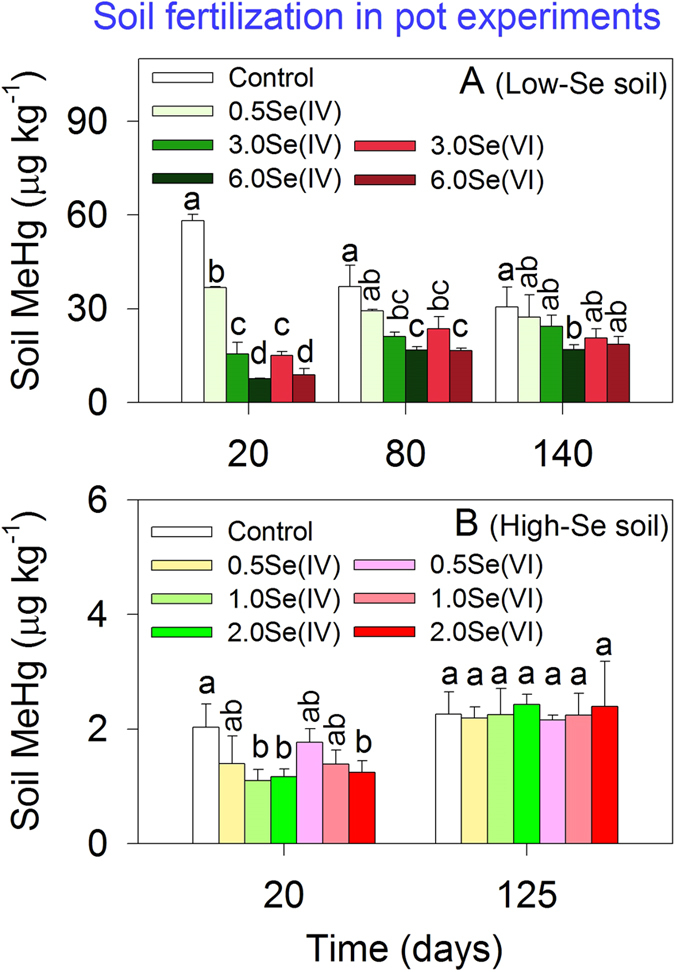
MeHg concentrations (means ± SD, n = 3) in soils under soil amendment with various levels of Se(IV) or Se(VI) in: (**A**) Low-Se soil and (**B**) High-Se soil. Different letters indicate significant differences among treatments within the same day (*p* < 0.05).

Soil MeHg concentrations in pot experiments exhibited temporal variation (Fig. 1). Both soils generally showed a decline in MeHg concentrations relative to the control under soil fertilization with Se, with a marked decrease by the first sampling day (day 20) and a less variation thereafter (Fig. 1). Typically, MeHg concentrations in the Low-Se soil decreased by 37–87% on day 20, 21–55% on day 80 and 10–44% on day 140 (Fig. 1A). Accordingly, lower MeHg concentrations under Se amendment were observed in porewater on day 20 and day 140 (SI Fig. S2). Moreover, significant differences in soil MeHg levels were noted among treatments (separate one-way ANOVA followed by Turkey’s test for each sampling, F_5.12_ ≥ 4.090, *p* ≤ 0.021). Anyway, MeHg levels were affected by amended Se dose (separate two-way ANOVA for each sampling, F_3, 17_ ≥ 7.512, *p* ≤ 0.003) but not Se species (F_1, 19_ ≥ 0.083, *p* ≥ 0.684). A similar scenario of MeHg dynamics was observed in the High-Se soil. MeHg levels decreased by 13–46% on day 20 and were less variable on day 125 (Fig. 1B), with Se dose having a significant effect on soil MeHg (two-way ANOVA, F_3, 20_ = 11.536, *p* < 0.0001) but not Se species (F_1, 22_ = 2.663, *p* = 0.122) on day 20.

**Figure 2:**
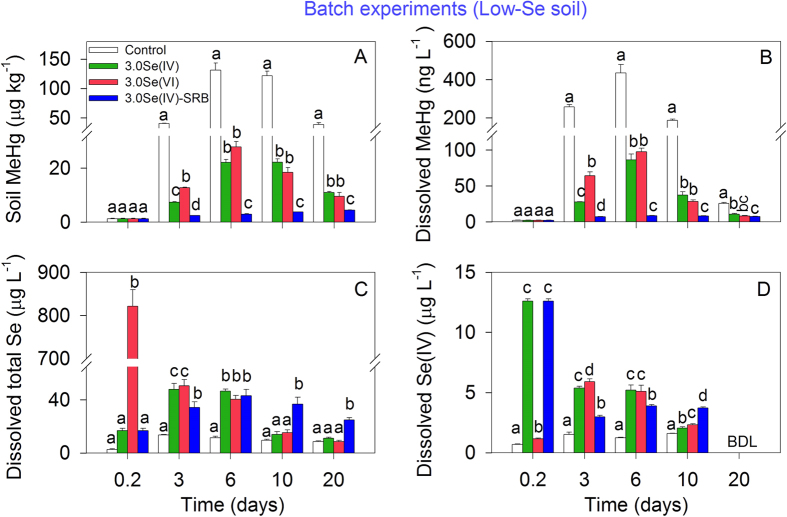
(**A**) MeHg concentrations in soils, (**B**) dissolved MeHg in overlying water, (**C**) dissolved total Se and (**D**) dissolved Se(IV) concentrations under soil fertilization with 3.0 mg kg^–1^ Se(IV), Se(VI), or Se(IV) with sulfate-reducing bacteria inhibitor (3.0Se(IV)-SRB) in batch experiments. BDL: Below detection limit. Data are given as means ± SD (n = 3). Different letters indicate significant differences among treatments within the same day (*p* < 0.05).

**Figure 3:**
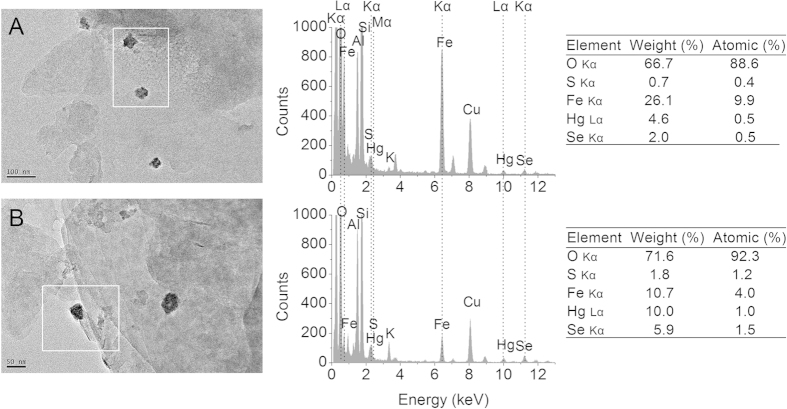
TEM images and EDX spectra of nanoparticles from the Low-Se soil spiked with 100 mg Hg kg^–1^ and 150 mg Se kg^–1^ after 20 days of incubation. The copper signal resulted from the copper grid. The oxygen, aluminum, silicon, potassium and part of iron resulted from the clay particles. **(A)** Scale bar = 100 nm; **(B)** scale bar = 50 nm.

**Figure 4:**
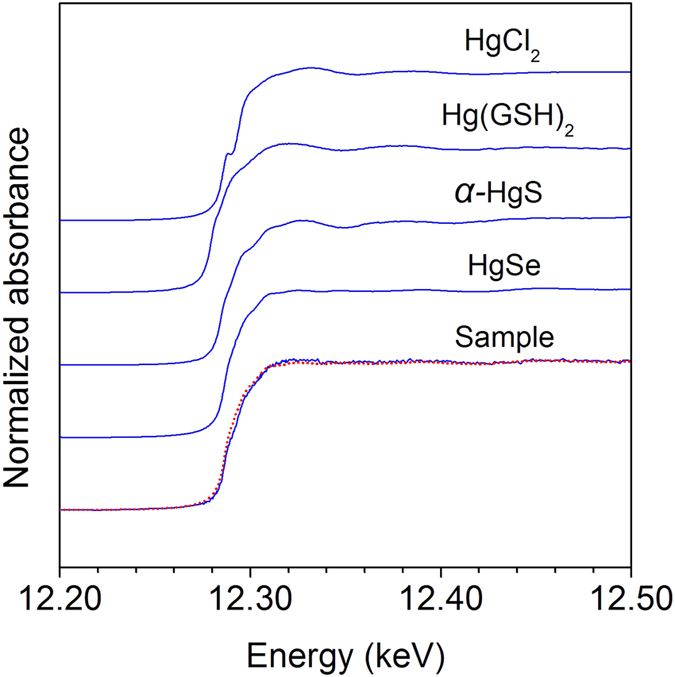
Hg L_III_ XANES spectra of the soil sample (100 mg Hg kg^–1^ and 150 mg Se kg^–1^) and the reference compounds (HgCl_2_, *α*-HgS, Hg-Glutathione (RS-Hg-SR) and HgSe). The red dot line demonstrated the highly consistent of the XANES spectrum of soil sample with that of HgSe compound.

Reduced soil and porewater MeHg concentrations under Se amendment were also observed in batch experiments. Methylmercury concentrations in the soil (Fig. 2A) and overlying water (Fig. 2B) in the batch experiments on day 20 closely resembled those observed in the pot experiment on the same day. Typically, the extent of the decrease in soil MeHg concentrations observed in the batch experiment on day 20 were 3.5 and 4.0 times lower for 3.0Se(IV) and 3.0Se(VI) treatments, respectively (Fig. 2A), comparable to the decreases of 3.8 and 4.0 times observed in their corresponding treatments in the pot experiment (i.e., 3.0Se(IV) and 3.0Se(VI); Fig. 1A). In addition, lower soil or dissolved MeHg levels in 3.0Se(IV)-SRB than those in the control (Fig. 2A,B) or control-SRB (SI Fig. S3), along with the temporal changes in dissolved total Se and Se(IV) concentrations (Fig. 2C,D), revealed that SRB and Se transformation may contribute to Se-induced reduction in soil MeHg levels (i.e., MeHg-Se antagonism in the soil) which would be discussed below. Transmission electron microscopy (TEM) and energy-dispersive X-ray spectroscopy (EDX) was conducted to obtain insight into the MeHg-Se antagonism in the soil. Similar to the pot and batch experiments, we also found that soil MeHg concentrations decreased significantly (from 184.5 to 12.8 μg kg^–1^) under Se amendment. TEM examination of this soil revealed nanoscale particles (~30 nm), closely associated clay particles (Fig. 3). EDX analysis confirmed that these nanoparticles contained Hg and Se with an approximate molar Hg:Se ratio of 1:1 (Fig. 3), indicative of Hg-Se complex forms. The soil used for TEM-EDX analysis was also measured by Hg L_III_-edge synchrotron radiation X-ray absorption near edge structure (XANES). The Hg L_III_-edge XANES spectrum of the sample was very similar to that of HgSe (Fig. 4), further evidencing that the solid-phase Hg speciation in the Se-amended soil could be dominated by HgSe.

### Accumulation of Se and MeHg in plant tissues under soil fertilization of Se

**Figure 5:**
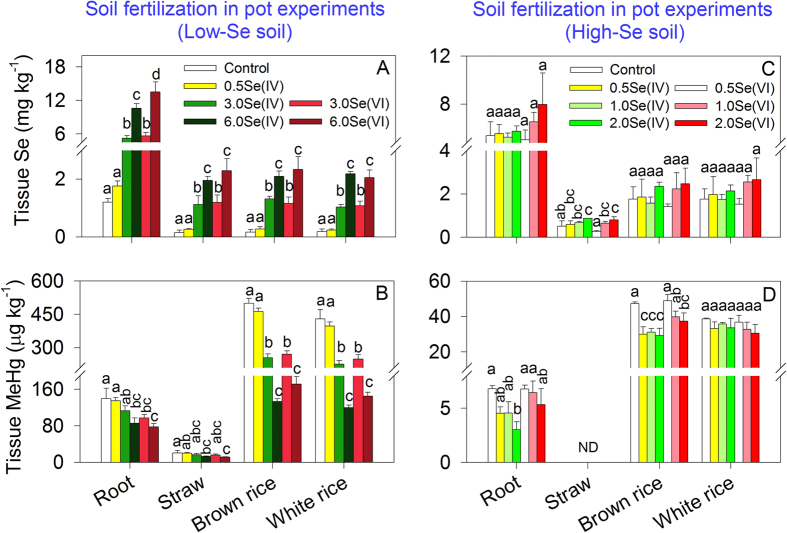
Tissue concentrations (root, straw, brown rice or white rice) of Se and MeHg following soil fertilization with Se. (**A**) [Se], Low-Se soil, (**B**) [MeHg], Low-Se soil, (**C**) [Se], Hihg-Se soil. (**D**) [MeHg], High-Se soil. Data are given as means ± SD (n = 3). ND: not detected. Different letters indicate significant differences among treatments within the same day (*p* < 0.05).

Concentrations of Se rose significantly in most tissues (except 0.5Se(IV) treatment) under soil fertilization with Se in the Low-Se soil (Fig. 5A, separate one-way ANOVA for each tissue, F_5, 12_ ≥ 38.148, *p* < 0.0001). Accompanying the increase in tissue Se levels, tissue MeHg concentrations declined significantly in the Low-Se soil (Fig. 5B, separate one-way ANOVA for each tissue, F_5, 12_ ≥ 5.236, *p* ≤ 0.009), especially in soils amended with higher doses of Se. Concentrations of MeHg decreased by 3–44% in root, 3–44% in straw, 7–73% in brown rice and 8–72% in white rice relative to the control. The decline in tissue MeHg was affected by Se dose (separate two-way ANOVA, F_3, 17_ ≥ 8.672, *p* ≤ 0.001) rather than Se species (F_1, 19_ ≤ 4.655, *p* ≥ 0.05). There appeared to be a less pronounced increase in tissue Se levels in the High-Se soil (Fig. 5C) as compared to the Low-Se soil (Fig. 5A): significant increases were only observed in straw at higher doses of Se amendment (i.e., 2.0Se(IV) and 2.0Se(VI)). In the case of High-Se soil, soil fertilization of Se resulted in 0.2–55%, 3–38%, or 5–21% decrease of MeHg levels in root, brown rice, or white rice (Fig. 5D). Both Se dose (separate two-way ANOVA, F_3, 20_ ≥ 6.532, *p* ≤ 0.004) and Se species (F_1, 22_ ≥ 19.435, *p* < 0.0001) influenced MeHg concentrations in root and brown rice. Furthermore, MeHg concentrations in brown and white rice were related negatively to soil Se concentrations when all data for High-Se and Low-Se soils were combined together (SI Fig. S4), suggesting the antagonistic effect of soil Se on grain MeHg accumulation. However, this inhibitory response did not result in a generalized toxic effect, as grain yield was unaffected by Se amendment (Table 2).

Methylmercury concentrated in rice grain within plants in Low-Se soil (85–92%, SI Fig. S5A) and MeHg accounted for 83% and 76% of the total mercury in brown rice and white rice, respectively (SI Fig. S6). Distribution of MeHg (%MeHg) in root and straw was significantly affected by soil fertilization with Se (SI Fig. S5A). Se amendment increased MeHg distribution in straw and root (separate one-way ANOVA, F_5, 12_ ≥ 7.816, *p* ≤ 0.002), but decreased distribution in grain (85–92% in Se-amended treatments compared to 92% in the control, one-way ANOVA, F_5, 12_ = 14.159, *p* < 0.0001). By comparison, Se distributed relatively evenly in grain, straw and root and was less affected by soil fertilization with Se (separate one-way ANOVA, F_5, 12_ ≤ 1.039, *p* > 0.05; SI Fig. S5B). Concentrations of MeHg in straw from the High-Se soil were too low to be precisely measured: total MeHg contents in digested straw samples were generally lower than 0.1 pg per 0.1 mL (maximum sample volume for MeHg analyzer), while the minimum detection level for MeHg was 0.1 pg. Accordingly the MeHg distribution was not calculated.

### Accumulation of Se and MeHg in plant tissues under foliar fertilization

Foliar fertilization with Se produced considerable increases in Se concentrations (separate one-way ANOVA, F_4, 10_ ≥ 28.418, *p* < 0.0001; SI Fig. S7A) in straw (5–11 fold), brown rice (3–12 fold) and white rice (3–15 fold), but not in root or soil (separate one-way ANOVA, F_4, 10_ ≤ 1.309, *p* ≥ 0.331). However, the corresponding MeHg levels were comparable among treatments (separate one-way ANOVA, F_4, 10_ ≤ 2.454, *p* ≥ 0.114; SI Fig. S7B). Therefore, foliar fertilization did not decrease MeHg accumulation in rice. And MeHg distribution in grain, straw and root was not significantly affected by foliar Se fertilization (separate one-way ANOVA, F_4, 10_ ≤ 0.519, *p* ≥ 0.724; SI Fig. S5C).

## Discussion

The elevated levels of MeHg in rice grain at mining and industrial sites have evoked public health concerns^2^,^26^. And increasing evidence from this (Fig. 5) and other studies^16^,^17^ indicate that Se in soil inhibits MeHg accumulation in rice grain. However, the mechanism underlying the inhibition of MeHg accumulation in rice plants is largely unknown, compared to the well-reported protective effect of Se on IHg accumulation in plants^9^,^11^,^12^,^13^,^14^.

The results from both pot and batch experiments demonstrate that soil MeHg levels decreased under soil fertilization with Se (Figs 1 and 2A), clearly indicating the inhibitory effects of Se amendment on soil MeHg levels. And the mechanisms were further explored below.

Firstly, Se transformation in soil and the formation of Hg-Se complexes observed in this study may be mainly responsible for the reduced soil MeHg concentrations under Se amendment. In the batch experiments, soil MeHg levels in 3.0Se(IV)-SRB (with SRB inhibitor) were significantly lower than those in 3.0Se(IV) treatment (Fig. 2A). This indicates that Se-induced decline in soil MeHg levels may be linked with microbial activities (e.g., mercury methylation and/or demethylation processes, primarily mediated by SRB^27^,^28^). Under anoxic or suboxic conditions in both batch and pot experiments (Eh = –232 to 58 mV), Se in soil could be microbially transformed (e.g., to selenide, elemental Se and/or organic Se^5^,^29^,^30^), considering that Se(IV) represents only 9–16% of total dissolved Se in all treatments, irrespective of initially added Se species (selenite or selenate, Fig. 2D). These Se species are hypothesized to reduce soil MeHg concentrations through modifying IHg speciation. For instance, Se may thermodynamically react with IHg to produce HgSe solid form (log K_sp_ = –58), which is less bioavailable for microbial methylation^19^,^31^. This is confirmed by our TEM-EDX analysis, which demonstrates nanoparticles containing Hg and Se with an approximate molar Hg:Se ratio of 1:1 (Fig. 3). Although the molar ratios of Hg:S in the nanoparticles was also close to 1:1, the Hg L_III_-edge XANES spectrum of the sample exhibited the typical spectral feature of HgSe, but not *α*-HgS (Fig. 4). Principal component analysis (PCA) also revealed that the type of probable species contained in the soil was dominated by HgSe rather than HgCl_2_, *α*-HgS or Hg-Glutathione (RS-Hg-SR). These results further confirm the formation of HgSe in the soil. Notwithstanding, other evidence also points to the inhibitory effects of Se on mercury methylation in sediment^32^ and bacterial cultures^18^,^19^, possible due to the formation of HgSe. However, the lower soil MeHg levels in 3.0Se(IV)-SRB treatment than control-SRB in the presence of SRB inhibitor (SI Fig. S3A) may indicate that other Hg-Se reactions, e.g., MeHg demethylation and/or volatilization aided by the addition of Se (similar to sulfide stimulated demethylation)^33^, could also partly account for the Se-induced reduction in soil MeHg levels.

Secondly, Se(IV) and Se(VI) were found to be equally effective in reducing MeHg concentrations in soils (Low-Se or High-Se, Figs 1 and 2A), which could also be due to Se transformation in soil under anoxic conditions. Initially, dissolved total Se and Se(IV) levels in the overlying water differed distinctly between 3.0Se(IV) and 3.0Se(VI) treatments (Fig. 2C,D). However, both total Se and Se(IV) concentrations became comparable between treatments by day 3, suggesting transformation of Se. In particular, a sharp increase in dissolved Se(IV) concentrations was observed in the 3.0Se(VI) treatment from day 0 to 3 (Fig. 2D), suggesting rapid conversion of Se(VI) to Se(IV). Therefore, Se transformation may explain why Se-induced reduction in soil MeHg levels is less dependent on the added Se species (i.e., Se(IV) or Se(VI)).

**Figure 6:**
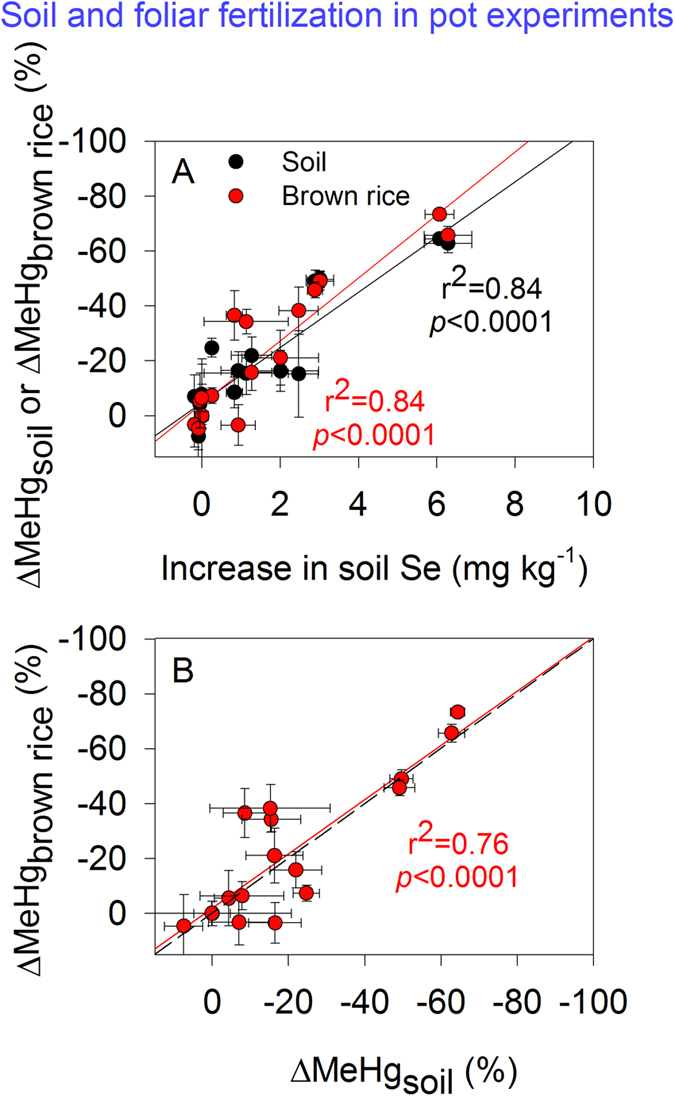
(**A**) Changes in MeHg concentrations in soil (ΔMeHg_soil_, %) and brown rice (ΔMeHg_brown rice_, %) as a function of the increase in soil Se concentrations following Se amendment (mg kg^–1^). (**B**) Changes in MeHg concentrations in brown rice (ΔMeHg_brown rice_, %) as a function of mean soil concentrations (ΔMeHg_soil_, %). Data from ‘soil fertilization’ experiments (High-Se and Low-Se soils) and ‘foliar fertilization’ experiments are included. Data are given as means ± SD (n = 3).

Thirdly, the potential effects of soil ambient Se on MeHg-Se antagonism were evaluated by comparing Low-Se soil and High-Se soil. Large differences in soil Se concentrations in mercury-contaminated areas have been documented (e.g., 0.16–36.6 mg kg^–1^ in Wanshan mercury mining area, China^16^). Ambient Se could be relatively refractory, compared to Se added as an amendment^34^. Therefore, it is necessary to explore potential effects of ambient Se levels on MeHg bioaccumulation in combination with Se amendment. The inhibitory effect of ambient Se in soil on grain MeHg levels has been reported recently in field-collected samples^16^. However in our study, addition of 0.5 mg Se(IV) kg^–1^ resulted in comparable reduction in soil MeHg levels in Low-Se and High-Se soils (37% and 31%, compared to the control) on day 20 (Fig. 1), despite of 11.6-fold difference in soil ambient Se levels. Meanwhile, variations in soil MeHg levels of either Low-Se or High-Se soil were found to be significantly correlated to the increase in soil Se levels (Fig. 6A, more details described below), possibly suggesting that amended Se may play a more important role in controlling soil MeHg levels in this study. The ambient soil Se in the mercury-mining area (from which the High-Se soil was sampled) is mainly in the form of sulfide-bound Se and residual Se, with an extremely low proportion as mobile Se (i.e., 2–3% as water-soluble and ligand-exchangeable Se)^34^. It is possible that ambient soil Se, containing more ‘aged’ forms of Se, may have a lower impact on the antagonism in soil compared to freshly added Se.

The results from our pot experiments clearly indicate that declines in soil MeHg levels and related plant uptake (11–71%, SI Fig. S8) ultimately result in reduction in grain MeHg concentrations (Fig. 5B,D). Therefore, the controls of ‘MeHg-Se antagonism in soil’ (i.e., Se-induced reduction in soil MeHg levels) on grain MeHg concentrations are further explored. A comparison between the changes in the concentration of MeHg (ΔMeHg, %) in soil and brown rice can be determined using:


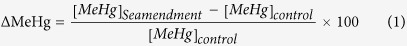


where [MeHg] is the MeHg concentration in brown rice (μg kg^–1^) or soil (μg kg^–1^). In view of the temporal variation in soil MeHg concentrations and the fact that soil MeHg bioaccumulates through plant growth^26^, soil MeHg levels were averaged over time to indicate exposure of rice plants to soil-MeHg during the whole growth period^35^.

The contribution of MeHg-Se antagonism in soil to the reduced plant MeHg accumulation is demonstrated by plotting these variations as a function of the increased Se concentrations in the soil following Se amendment (Fig. 6). For both High-Se and Low-Se soils, variations in brown rice and soil MeHg levels exhibited similar declining trends with increasing soil Se and less differentiation between the curves was noted (Fig. 6A), emphasizing that decreased grain MeHg levels could mostly be attributed to reduced soil MeHg levels. Moreover, the correlation between changes in MeHg levels in brown rice (ΔMeHg_brown rice_) and those in soil (ΔMeHg_soil_) is significant (*p* < 0.0001; Fig. 6B), closely following a 1:1 relationship for all data (both High-Se and Low-Se soils), implying that the role of MeHg-Se antagonism in decreasing grain MeHg concentrations is crucial.

The critical role of antagonism in soil is further evidenced by foliar fertilization. Whereas soil fertilization with Se would result in antagonism within soil and plant (if they exist), foliar fertilization would enhance only tissue concentrations of Se and produce antagonism within plant. Our results show that foliar fertilization with Se increased grain and straw concentrations of Se (SI Fig. S7A,) similar to those observed under soil fertilization in Low-Se soil (Fig. 5A). In contrast, under foliar fertilization with Se, MeHg levels in grain, straw and root (SI Fig. S7B), together with MeHg uptake in plant (SI Fig. S8), did not differ significantly among Se-amended treatments and the control. This suggests that elevated Se in aboveground tissues may not be responsible for the lower grain MeHg levels, i.e., antagonism within plant has a marginal effect on MeHg bioaccumulation. This is also the case for soil fertilization with Se(IV) in the High-Se soil. The inhibition of MeHg accumulation in grain from High-Se soil (decrease of 34–38%, *p* < 0.01) is unlikely to be derived mainly from the accumulated Se within plant but rather from antagonism in soil, because only a small and insignificant increase in tissue Se levels (*p* > 0.05) was observed (Fig. 5C).

Additional evidence about the dominance of MeHg-Se antagonism in soil over antagonism within plant can be obtained by comparing MeHg and Se distribution within plant. If antagonistic interaction between Se and MeHg within plants plays a key role in reducing grain MeHg levels, we would expect a similar pattern of MeHg distribution among the tissues following that of Se; however, the majority of MeHg is found in the grain (85–92%) whereas Se is evenly distributed in the plant (SI Fig. S5B). A recent study using synchrotron radiation microscopic X-ray fluorescence (SR-μXRF) also reveals different distribution patterns of Hg and Se in brown rice, suggesting decoupling of Hg and Se in grain^36^.

Although MeHg-Se antagonism within soil is most likely dominant, MeHg-Se antagonism within plant could not be completely ruled out, as indicated by the significant changes in MeHg distribution (up to ~8%, compared to averagely 29% decrease in soil MeHg levels) under soil fertilization with Se; the addition of Se resulted in a proportional increase in MeHg distribution in root and straw, and a corresponding decrease in brown rice (SI Fig. S5A).

The lower proportion of grain MeHg under soil Se fertilization relative to the control could be unlikely the consequence of the formation of inert MeHg-Se complexes in the aboveground tissues or *in vivo* demethylation of MeHg facilitated by Se amendment within plant, given that foliar fertilization elevated Se levels in the aboveground tissues but did not reduce the corresponding MeHg concentrations. Instead, we propose that the enhanced MeHg distribution in root under soil fertilization could possibly be due to the MeHg-Se interaction within rice root (e.g., formation of MeHg-Se complexes), which needs to be confirmed in future studies, e.g., by analyzing changes in MeHg speciation and Se-containing proteins within root under Se amendment. However, changes in MeHg distribution in root and thus MeHg-Se antagonism were not observed under foliar fertilization (SI Fig. S5C), possibly because of the relatively constant Se levels in root under foliar fertilization. Alternatively, the node, a junctional area of leaves and branches to the stem, may simply regulate MeHg distribution under Se amendment, as has been demonstrated for Cd, Mn and Zn^37^. Further researches are warranted.

Selenium fertilizer has been proposed as an effective strategy for enhancing Se levels in crops and for increasing dietary Se intake in Se-deficient populations^38^,^39^. This is based on the fact that, in China, plant-based food is the major dietary source of Se^38^ and that most rice has a Se concentration insufficient for nutritional requirements^40^. However, foliar fertilization appears to be less effective than soil fertilization at decreasing MeHg availability and plant uptake. More knowledge about transport channels and speciation of Se under foliar and soil fertilization would help explain the observed phenomena.

### Conclusions and Implications

We demonstrate that the amendment of Se into soil decreases soil MeHg concentrations, which is well in line with earlier reports^17^,^41^. By providing multiple lines of evidence (i.e., formation of IHg-Se complexes in soils, coupling of reduction in soil and grain MeHg levels, distinct distribution of MeHg and Se within plants, and constant grain MeHg levels under foliar application with Se), we propose that the antagonism in soil (i.e., Se-induced reduction in soil MeHg levels) might be the key process triggering the reduction in grain MeHg levels; alternately, MeHg-Se antagonism within plant is probably not sufficient to induce these reductions. These findings highlight the weakness in some hydroponic studies where only antagonism within the plant was addressed. Furthermore, the previous use of oxic media and short-term exposures^10^ may have obscured the effects of Se on soil MeHg levels. Possible mechanisms of reduced MeHg bioaccumulation under Se amendment in soil-rice systems were summarized in SI Fig. S9.

The present study provides data that elucidate how Se species, Se doses and ambient Se levels in soil affect the MeHg accumulation in rice. Both species of Se, i.e., selenite and selenate, were equally effective in decreasing soil MeHg in both High-Se and Low-Se soils. Increasing the Se dose was effective in reducing MeHg concentrations in grain, however, concerns about potential detrimental effects of Se should be considered due to the narrow range between Se deficiency and toxicity to plants. The highest Se level measured in the straw was 2.3 mg Se kg^–1^ (6.0Se(VI) in Low-Se soil), which did not reduce crop yield (Table 2) and was within the toxicity threshold for rice^42^. Although elevated levels of Se raise concerns about potential selenium toxicity to the plants and their consumers, nevertheless, fertilization of soil with Se is recommended to meet the Se needs of growing plants while simultaneously decreasing MeHg accumulation. Moreover, the results indicate that Se fertilization approaches and Se doses largely influence MeHg-Se antagonism. Thus, antagonism in soil and its influencing factors should be carefully considered when assessing bioavailability and the subsequent risk/toxicity of mercury in contaminated soil.

## Methods

### Soils

Two paddy soils, namely Low-Se and High-Se, were used in the study. The large differences in Se concentrations in the two soils enabled the investigation of potential effects of ambient Se concentrations on MeHg-Se antagonism. Detailed information on soil sampling and preparation as well as soil characteristics are given in Table 1.

### Selenium fertilization via soil and foliar application

Two sets of experiments, i.e., ‘soil fertilization’ (pot or batch experiment) using both Low-Se and High-Se soils and ‘foliar fertilization’ (pot experiment) using Low-Se soil only, were conducted (Table 2). In ‘soil fertilization’ experiments, sodium selenite (Se(IV)) or selenate (Se(VI)) (40 mg Se L^–1^; Sigma Aldrich) was mixed thoroughly with the soils to reach a range of environmentally relevant Se concentrations^39^,^43^. The Se doses (i.e., 0–6 mg kg^–1^ Se for Low-Se soil and 0–2 mg kg^–1^ Se for High-Se soil) were chosen to achieve Se-sufficiency but not toxicity to the rice plants^44^. Because of the relatively high ambient Se concentration in the High-Se soil and to preclude potential Se toxicity, lower Se doses were amended into the High-Se soil, compared to the Low-Se soil.

In ‘foliar fertilization’ experiments, Se(IV) or Se(VI) (the same as ‘soil fertilization’) was sprayed carefully onto leaves using aerosol sprayers on day 60 (stem extension stage) and day 80 (heading stage) for the Low-Se soil only. Two application rates, i.e., 30 g and 80 g Se ha^–1^ (0.27 mg and 0.72 mg Se pot^–1^), were used, resulting in four treatments, namely Se(IV)-low application rate (AR), Se(IV)-high AR, Se(VI)-low AR and Se(VI)-high AR, plus the control (Table 2). The AR’s were similar to rates reported previously^45^. In addition, results from preliminary experiments indicated these AR’s would produce tissue Se concentrations comparable to those obtained under soil fertilization, facilitating direct comparison between soil fertilization and foliar fertilization. The soil was covered with plastic wrap and pots were separated from other treatments to avoid cross-contamination during spraying.

### Pot experiments

Pot experiments were completely randomized with a factorial arrangement of the treatments: two fertilization approaches (soil and foliar fertilization), two soil types (Low-Se and High-Se soils) and two Se species (Se(IV) and Se(VI)) were tested, resulting in a total of 51 pots for 17 treatments (triplicates for each treatment; see Table 2).

For all treatments (i.e., control and Se-amended treatments), soils were first flooded with deionized water and equilibrated for 20 days. Rice seeds (*Oryza sativa* L., *indica*) were germinated and then cultivated in a mixture of peat soil (Se 0.56 ± 0.06 mg/kg THg; 93.4 ± 10.5 μg/kg; MeHg 0.10 ± 0.05 μg/kg), vermiculite and perlite (3:2:1, v/v/v) for 30 days at ambient temperature, prior to transplant. And thirty day-old seedlings of rice were then transplanted into 6 L plastic pots filled with 2.5 kg soil. Selenium-free granulated fertilizer (0.27 g kg^–1^ Ca(HPO_4_)_2_·H_2_O, 0.24 g kg^–1 ^KCl, and 0.30 g kg^–1^ CO(NH_2_)_2_) was applied to the pots prior to transplanting; this treatment was repeated on day 50 (stem extension) and day 80 (heading stage). Overall, 79 mg P kg^–1^, 150 mg K kg^–1^ and 167 mg N kg^–1^ were supplied to each pot over the course of the experiment. Plants were grown at ambient temperature (15–38 ^o^C) under flooded conditions (2 cm deionized water above soil surface) until final harvest at physiological maturity (125-d for High-Se soil or 140-d for Low-Se soil). Soil redox potential was also monitored. Water was drained from the pots just prior to harvest.

Surface soils (1–11 cm) were sampled on day 20 (initial seedling transplants) and day 125 (harvest) for High-Se soil, and on day 20 (seedling transplants), day 80 (heading stage) and day 140 (harvest) for Low-Se soil. All soil samples were vacuum-packed immediately, placed in an ice box and transferred to the laboratory within 3 h of sampling. Porewater was collected by centrifuging soil subsamples at 2000 × g and filtering through 0.45 μm polyethersulfone filter capsules (Anpel, China). Porewater was preserved with 0.4% HCl (v/v) and stored, together with the soil samples, at –20 ^o^C, until MeHg analysis. Sample preparation was conducted in a glove bag (AtmosBag, Sigma Aldrich) filled with high-purity N_2_ (99.999%) unless otherwise specified.

At harvest, plants were rinsed thoroughly with deionized water and separated into grain, straw and roots. Iron plaque on roots was removed as previously described^46^. After freeze drying (Labconco, USA), straw and roots were ground to a fine powder with an IKA basic analytical mill (IKA A11, Germany). Husks were removed manually from grain (brown rice) and subsamples were milled using a benchtop rice polisher to produce ‘white rice’, freeze-dried and ground into ≤ 0.15 mm powder. All plant tissue samples were kept at –80 ^o^C until further analysis.

### Batch experiments

To further explore the earlier (day 0 to 20) probably more dynamic phases of MeHg-Se antagonism in soils (i.e., Se-induced reduction in soil MeHg levels), batch experiments were performed in the laboratory using 50 mL acid-clean centrifuge tubes (Corning, Mexico) as batch reactors (triplicates for each time-point). Moreover, conducting these batch experiments precluded the need to intensively sample soil from pots during the early phase of experiment, which may have altered soil conditions, and concentrations and availability of MeHg. Briefly, 10 g of air-dried Low-Se soil, spiked with Hg and Se at concentrations identical to those used in the pot experiment (i.e., 2.0 mg Hg kg^–1^ and 3.0 mg Se(IV) or Se(VI) kg^–1^, Table 2), was flooded with 30 mL 18 Ω water. All soils were amended with 10 mM Na-lactate as an electron donor prior to pre-incubation; treatments amended with 20 mM sodium molybdate as a SRB inhibitor (i.e., control-SRB) or amended with 3.0 mg Se(IV) kg^–1^ and 20 mM sodium molybdate (i.e., 3.0Se(IV)-SRB) were also involved. After pH adjustment to 5.5 (Table 1), the tubes were sealed, incubated in the dark at 28 ^o^C and mixed by turning the tubes end-over-end (manually) twice per day.

On day 0.2 (i.e., 4 h), 3, 6, 10 and 20, after redox potential (Eh) measurement using HQ30d multi-parameter meters (HACH, USA), samples were centrifuged (2000 × g) to separate soil and overlying water. For MeHg and Se(IV) analysis, subsamples of overlying water were preserved with 0.4% HCl (v/v) and stored at –20 ^o^C whereas for total Se determination, subsamples were preserved with 2% HNO_3_ (v/v) and stored at 4 ^o^C. Soil samples were frozen at –20 ^o^C until MeHg analysis.

In parallel, the Low-Se soil was also spiked with 100 mg Hg kg^–1^ and 150 mg Se kg^–1^ (the same Hg:Se ratio like those used in pot and batch experiments). Then the spiked soil was incubated for 20 days like above. Afterwards, the samples were centrifuged (2000 × g), and the solid was freeze-dried, mixed homogenously and sealed in a tube under N_2_ in a glove bag (AtmosBag, Aldrich). The formation of Hg-Se complexes in the soil was identified by using TEM equipped with EDX as well as Hg L_III_-edge XANES.

### Analytical methods and statistical analysis

Detailed information on analytical methods is given in the SI, including total Hg, MeHg and Se analysis of the porewater, soil and plant tissues, TEM-EDX and XANES. Certified reference materials, digestion blanks and matrix spikes were included for QA/QC as described in the SI (Table S1).

Statistical analyses were carried out (SPSS, version 16.0) using Tukey’s multiple comparison test of one-way analysis of variance (ANOVA) or two-tailed paired t tests (*p* < 0.05) to verify significant differences among treatments and using two-way ANOVA to assess the effects of Se dose and Se species on MeHg accumulation in plant tissues.

## Additional Information

**How to cite this article**: Wang, Y. *et al.* Mechanistic understanding of MeHg-Se antagonism in soil-rice systems: the key role of antagonism in soil. *Sci. Rep.* **6**, 19477; doi: 10.1038/srep19477 (2016).

## Supplementary Material

Supplementary Information

## Figures and Tables

**Table 1:** Characteristics of the Low-Se and High-Se soils in this study. Values are given as means ± SD (n = 3).

Soil characteristics	Units	Low-Se	High-Se
pH		5.5 ± 0.0	8.3 ± 0.2
Sand	%	6.7 ± 0.9	7.6 ± 2.3
Slit	%	86.2 ± 4.4	74.9 ± 1.1
Clay	%	7.1 ± 3.4	17.5 ± 1.2
TOC	%	2.1 ± 0.0	2.5 ± 0.3
Total Hg	mg kg^–1^	2.35 ± 0.15	41.55 ± 4.54
MeHg	μg kg^–1^	1.21 ± 0.20	2.02 ± 0.36
Total Se	mg kg^–1^	0.91 ± 0.10	10.55 ± 0.17

**Table 2:** Amended Se species/doses and crop yields (means ± SD, n = 3) following soil or foliar fertilization with Se.

	Soil	Treatments	Selenium doses	Crop yields (g pot^–1^dw) Brown rice
Soil fertilization	Low-Se	Control	0	22.9 ± 1.1
		0.5Se(IV)	0.5 mg selenite kg^–1^	22.2 ± 1.3
		3.0Se(IV)	3.0 mg selenite kg^–1^	20.6 ± 0.9
		6.0Se(IV)	6.0 mg selenite kg^–1^	22.8 ± 0.8
		3.0Se(VI)^a^	3.0 mg selenate kg^–1^	21.8 ± 1.3
		6.0Se(VI)	6.0 mg selenate kg^–1^	20.1 ± 1.0
	High-Se	Control	0	23.4 ± 2.1
		0.5Se(IV)	0.5 mg selenite kg^–1^	21.9 ± 2.1
		1.0Se(IV)	1.0 mg selenite kg^–1^	24.6 ± 4.7
		2.0Se(IV)	2.0 mg selenite kg^–1^	24.2 ± 4.7
		0.5Se(VI)	0.5 mg selenate kg^–1^	20.9 ± 1.5
		1.0Se(VI)	1.0 mg selenate kg^–1^	22.1 ± 4.6
		2.0Se(VI)	2.0 mg selenate kg^–1^	21.7 ± 1.9
Foliar fertilization	Low-Se	Se(IV)-low AR	30 g selenite ha^–1^	21.7 ± 1.3
		Se(IV)-high AR	80 g selenite ha^–1^	24.4 ± 0.7
		Se(VI)-low AR	30 g selenate ha^–1^	23.0 ± 3.5
		Se(VI)-high AR	80 g selenate ha^–1^	25.1 ± 1.2
Batch experiments	Low-Se	Control	0	NA
		Control-SRB	0 + SRB inhibitor	NA
		3.0Se(IV)	3.0 mg selenite kg^–1^	NA
		3.0Se(VI)	3.0 mg selenate kg^–1^	NA
		3.0Se(IV)-SRB	3.0 mg selenite kg^–1^ + SRB inhibitor	NA

^a^ SRB: sulfate-reducing bacteria. NA: not applicable.^a^0.5Se(VI) treatment was not included due to the limited availability of Low-Se soil.
