# Supplementary material for: Mechanistic understanding of MeHg-Se antagonism in soil-rice systems: the key role of antagonism in soil
Source: Sci Rep. 2016 Jan 18;6:19477. doi: 10.1038/srep19477 (PMC4726087; doi:10.1038/srep19477)
Supplement: Supplementary Information [file srep19477-s1.doc]

**Supplementary Information for**

**Mechanistic understanding of MeHg-Se antagonism in soil-rice systems: the key role of antagonism in soil**

Yongjie Wang^a#^, Fei Dang^b#^, R. Douglas Evans^a,c^, Huan Zhong^a,d*^, Jiating Zhao^e^ Dongmei Zhou^b^

^a^State Key Laboratory of Pollution Control and Resources Reuse, School of the Environment, Nanjing University, Nanjing 210023, P.R. China

^b^Key Laboratory of Soil Environment and Pollution Remediation, Institute of Soil Science, Chinese Academy of Sciences, Nanjing 210008, P.R. China

^c^Environmental and Resource Studies Program (ERS), Trent University, Peterborough, Ontario, Canada

^d^Environmental and Life Sciences Program (EnLS), Trent University, Peterborough, Ontario, Canada

^e^Key Lab for Biomedical Effects of Nanomaterial and Nanosafety, Laboratory of Metallomics and Metalloproteomics, Institute of High Energy Physics, Chinese Academy of Sciences, Beijing 100049, P.R. China

*^#^*These authors contributed equally.

*Corresponding author: Huan Zhong.

Tel: +86-25-89680316

Fax: +86-25-89680316

E-mail: zhonghuan@nju.edu.cn

**Content Page**

**1. Materials and methods S4**

1.1 Soils

1.2 Analysis methods of MeHg, total Hg and Se

1.3 TEM-EDX analysis

1.4 Hg L_III_-edge XANES analysis

**2. Tables S8**

Table S1 Certified reference materials and recovery rates

**3. Figures S9**

Figure S1. Soil Se concentrations following soil fertilization

Figure S2. Concentrations of MeHg in porewater in Low-Se soil

Figure S3. MeHg in soil and overlying water under SRB inhibitor amendment

Figure S4. Grain MeHg levels following soil fertilization with Se

Figure S5. Distribution of MeHg and Se in plant tissues

Figure S6. Relationships between MeHg and THg in brown rice and white rice

Figure S7. Se and MeHg concentrations in the soil and plant tissues following

foliar fertilization with Se

Figure S8. Total uptake of MeHg in rice plant of per pot following soil

fertilization with Se

Figure S9. Possible mechanisms of MeHg-Se antagonism in soil-rice systems

**4. References S18**

**1. Materials and methods**

**1.1 Soils**

High-Se soil, with a total Hg concentration of 41.55 ± 4.54 mg kg^–1^, was collected from a mercury contaminated paddy field in Wanshan, Guizhou Province of China. Low-Se soil, with a total Hg level of 0.18 ± 0.03 mg kg^–1^, was sampled in Yixing, Jiangsu Province of China. To ensure measurable mercury concentrations in rice plants, Low-Se soil was spiked with freshly prepared Hg(II) stock solution (80 mg L^–1^ as mercury nitrate monohydrate) to reach a concentration of 2.35 ± 0.15 mg total Hg kg^–1^ and mixed thoroughly. In this study, Hg(II) instead of MeHg was spiked into the Low-Se soil, in order to explore changes in net MeHg production in soils under Se amendment. The spiked soil was equilibrated for 20 days, after which changes in mercury solid speciation generally leveled off^1–3^. Both High-Se and Low-Se soils were homogenized, air-dried and sieved to an effective diameter of ≤ 2 mm before use. Soil characteristics were determined, including pH (by HQ30d, HACH, USA), total organic carbon (by vario TOC cube, Elementar, Germany), particle size (by LS 13320, Beckman Coulter, USA), total mercury (THg), methylmercury (MeHg) and selenium (Se) concentrations.

**1.2 Analysis methods of** **MeHg, total Hg and Se**

Plant tissues (0.02–0.05 g dw) were digested in 25% KOH/methanol (w/w) at 60 ^o^C for 4 h; soils (1.0–2.0 g wet weight) were extracted with HNO_3_/CuSO_4_-CH_2_Cl_2_, heated in a 45 ^o^C water bath for 45 min^4^, and purged (~10 min) with N_2_ (80–100 mL min^–1^) ^5^. All digests were stored at 4 ^o^C in the dark for less than 24 h before MeHg measurement.

Total mercury levels in the plant and soil digests were analyzed by thermal decomposition and atomic absorption spectrometry using a direct mercury analyzer (DMA-80, Milestone Scientific, Italy), according to USEPA Method 7473^6^. The minimum detection level (MDL) for THg was 0.005 ng. Concentrations of MeHg were measured using an automatic MeHg analyzer (Brooks Rand, USA) according to USEPA Method 1630^7^. The MDL for MeHg was 0.1 pg.

Selenium levels in plants, soils and overlying water were determined as previously reported^8^. Plant samples (0.02–0.08 g dw) were pre-digested with 1 mL HNO_3_ overnight and then heated at 120 ^o^C for 2 h. After cooling, 0.2 mL H_2_O_2_ was added and the mixture was heated at 90 ^o^C for 30 min. The soils were digested using a microwave system (Ethos EZ, Milestone Scientific, Italy) in HNO_3_ and HF (4:4 v/v) at 120 ^o^C for 2.5 h. All digests were filtered through a 0.45 µm filter (Anpel, China) and diluted to 5 mL. Se in soil and plant tissues was determined by inductively coupled plasma mass spectrometry (NexION-300 ICP-MS, PerkinElmer, USA) in collision cell mode with kinetic energy discrimination; indium (In) was used as an internal standard. Dissolved selenite concentrations in the overlying water (batch experiment) were analyzed by atomic fluorescence spectrometry AFS-230E (Haiguang, China).

Certified reference materials (Table S1), digestion blanks and matrix spikes were measured in parallel for quality control. Each sample was analyzed in duplicate whenever possible. All concentrations are reported on a dry weight basis. Soil water content was determined by drying the soils for 48 h at 105 ^o^C.

**1.3 TEM-EDX analysis**

For TEM-EDX analysis, the soil samples were dispersed in ethanol under ultrasonic treatment. And the suspension was dropped onto a 200 mesh copper grid coated with carbon film. Samples were imaged by a TEM (JEM-2100, JEOL, Japan) at an accelerating voltage of 200 kV. Elemental composition of nanoparticles in the selected area was determined by energy dispersive X-ray spectrometer (EDAX, USA) with a super-ultra thin window sapphire detector.

**1.4 Hg L_III_-edge XANES analysis**

Soil samples used for TEM-EDX examination were also used for XANES analysis at BL14W1 (3.5 GeV, 250 mA) beamline in Shanghai Synchrotron Radiation Facility (SSRF, China). The soils (≤ 0.063 mm) were prepared as a small pellet (1 mm thick). Energy was scanned from –200 to 450 eV relative to the Hg L_III_-edge (12,284 eV, HgCl_2_). The sample was scanned in fluorescence mode using a Si(111) double-crystal monochromator with a 19-element Ge solid detector at room temperature under air. To attenuate the interference of Fe Kα fluorescence from the sample, aluminum foils were set between the sample and the detector. Spectra of Hg reference compounds, including HgCl_2_, α-HgS, Hg-Glutathione (RS-Hg-SR) and HgSe, were recorded in transmission mode. After background subtraction and normalization, XANES spectrum for the soil sample was contrasted with possible Hg standard model compounds using ATHENA software^9^.

**2. Tables**

**Table S1**. Certified reference materials and the corresponding recovery rates. Data are given as means ± SD (n = 9).

| Certified reference materials | Source | Matrix | Element | Certified value | Measured value | Recovery  rate |
| --- | --- | --- | --- | --- | --- | --- |
|  |  |  |  |  |  |  |
|  |  |  |  | µg kg^–1^ | µg kg^–1^ | % |
| GBW07405 | NRCCRM^a^ | Soil | THg | 290 ± 30 | 298 ± 14 | 103 ± 5 |
| GBW10010 | NRCCRM | Rice | THg | 5.3 ± 0.5 | 5 ± 1 | 102 ± 13 |
| ERM- CC580 | IRMM^b^ | Estuarine | MeHg | 75 ± 4 | 68 ± 5 | 90 ± 7 |
|  |  | sediment |  |  |  |  |
| DORM-3 | NRCC^c^ | Fish muscle | MeHg | 355 ± 56 | 298 ± 20 | 84 ± 6 |
| GBW10010 | NRCCRM | Rice | Se | 61 ± 15 | 70 ± 16 | 108 ± 16 |
| GBW07405 | NRCCRM | Soil | Se | 1600 ± 200 | 1680 ± 201 | 105 ± 10 |

^a^ National Research Center for Certified Reference Materials, China.

^b^ Institute for Reference Materials and Measurements.

^c^ National Research Council Canada.

**3. Figures**

**Figure S1.** Soil Se concentrations following soil fertilization with Se in: A) Low-Se soil and B) High-Se soil. Data are given as means ± SD (n = 3). Different letters indicate significant differences among treatments within the same day (*p* < 0.05).


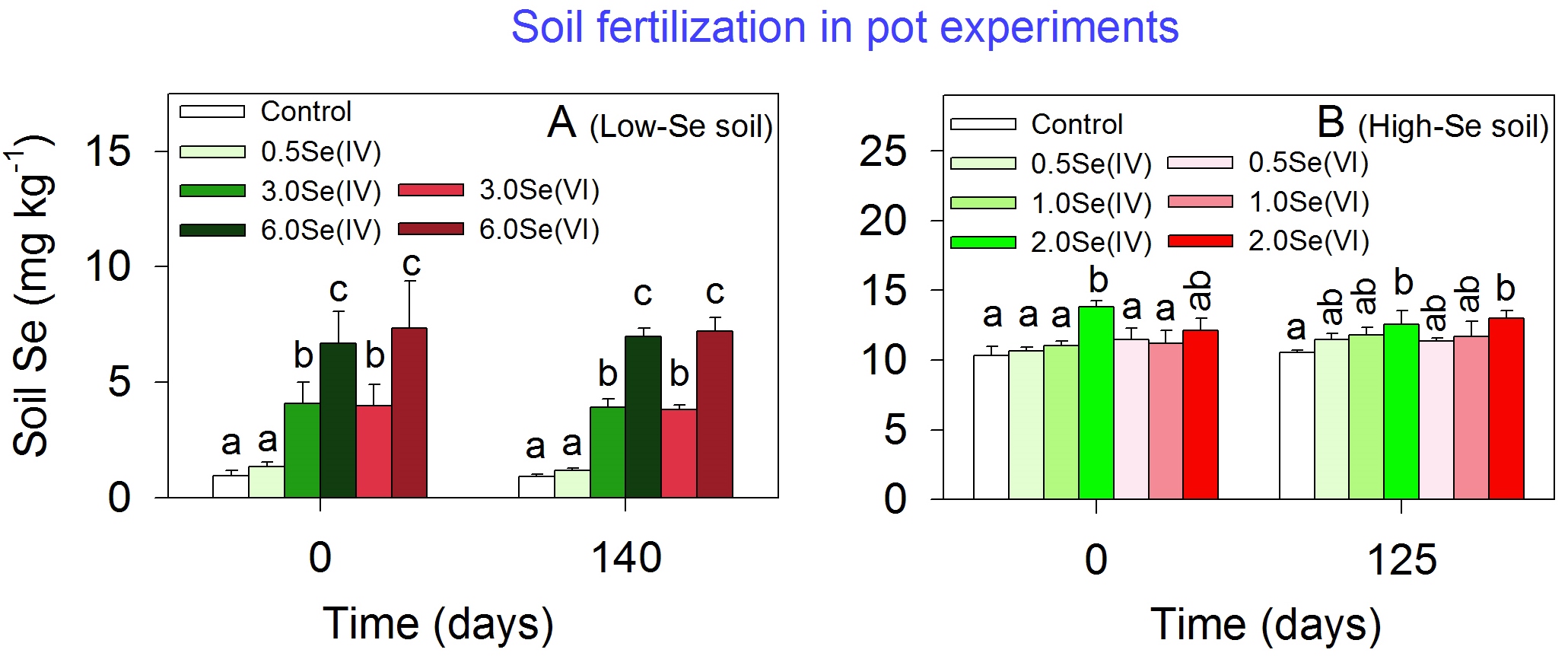


**Figure S2.** Concentrations of MeHg in porewater under Se amendment in Low-Se

soil. Data for High-Se soil are not available. Data are given as means ± SD (n = 3). Different letters indicate significant differences among treatments within the same day (*p* < 0.05).


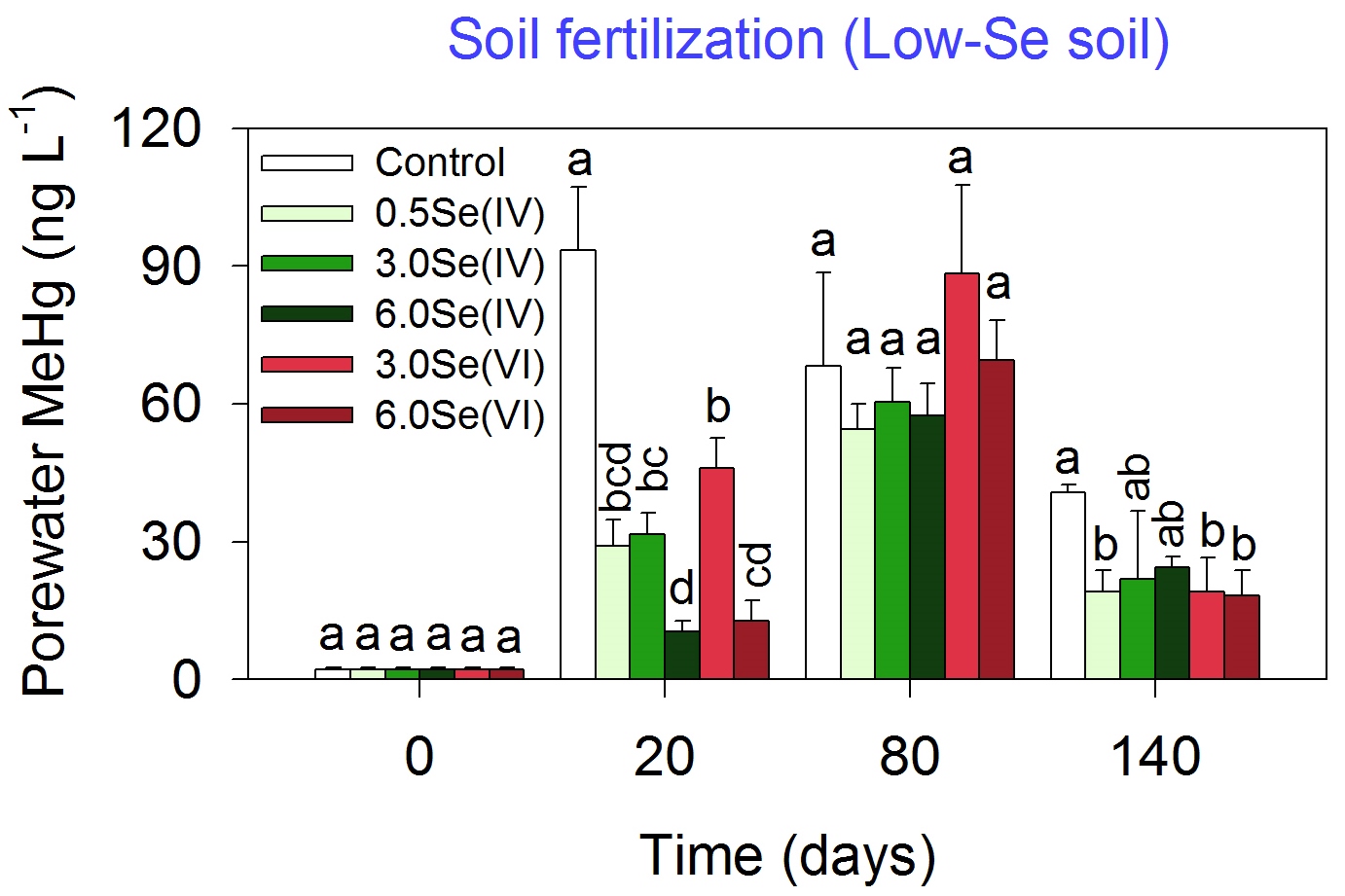


**Figure S3.** Concentrations of MeHg in soil (A) and overlying water (B) in treatments amended with sodium molybdate as sulfate-reducing bacteria (SRB) inhibitor (Control-SRB) or amended with 3.0 mg Se(IV) kg^–1^ and sodium molybdate (3.0Se(IV)-SRB). Data are given as means ± SD (n = 3). Different letters indicate significant differences between treatments within the same day (*p* < 0.05).


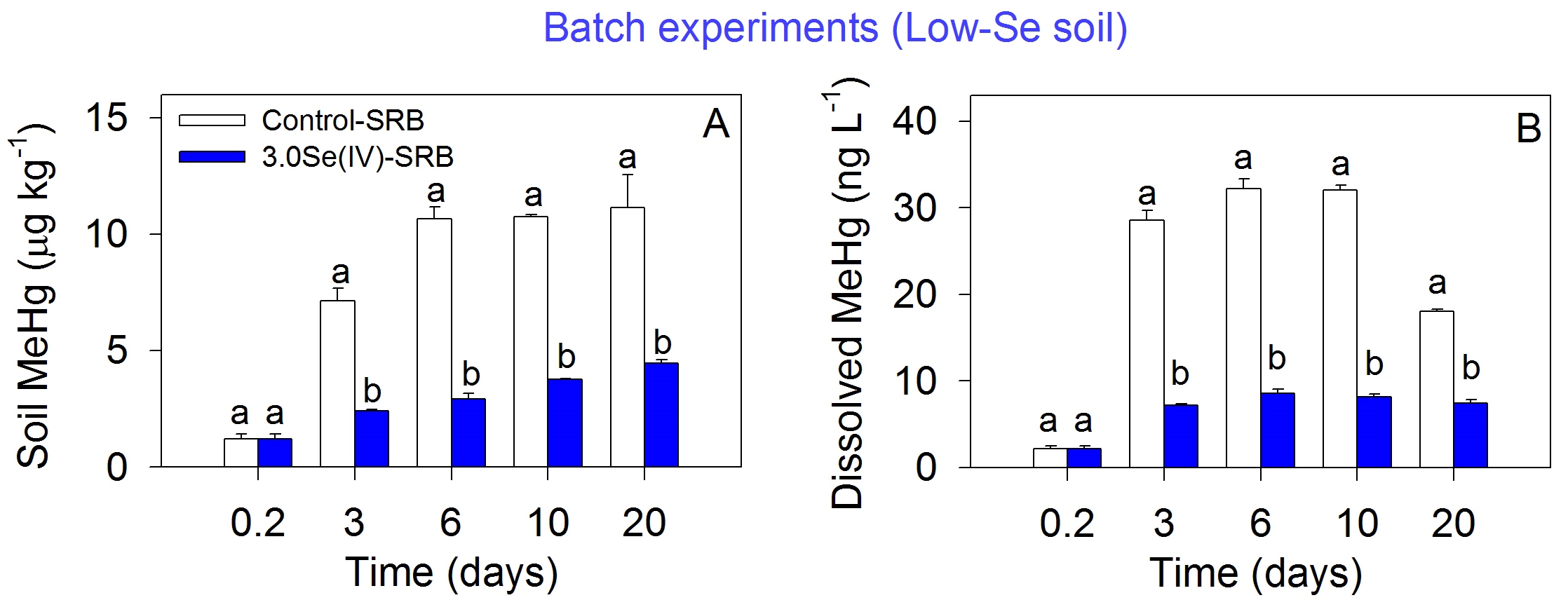


**Figure S4** A) MeHg concentrations in brown rice and B) white rice as a function of soil Se concentrations under Se amendment in both High-Se and Low-Se soils. Data are given as means ± SD (n = 3).

**
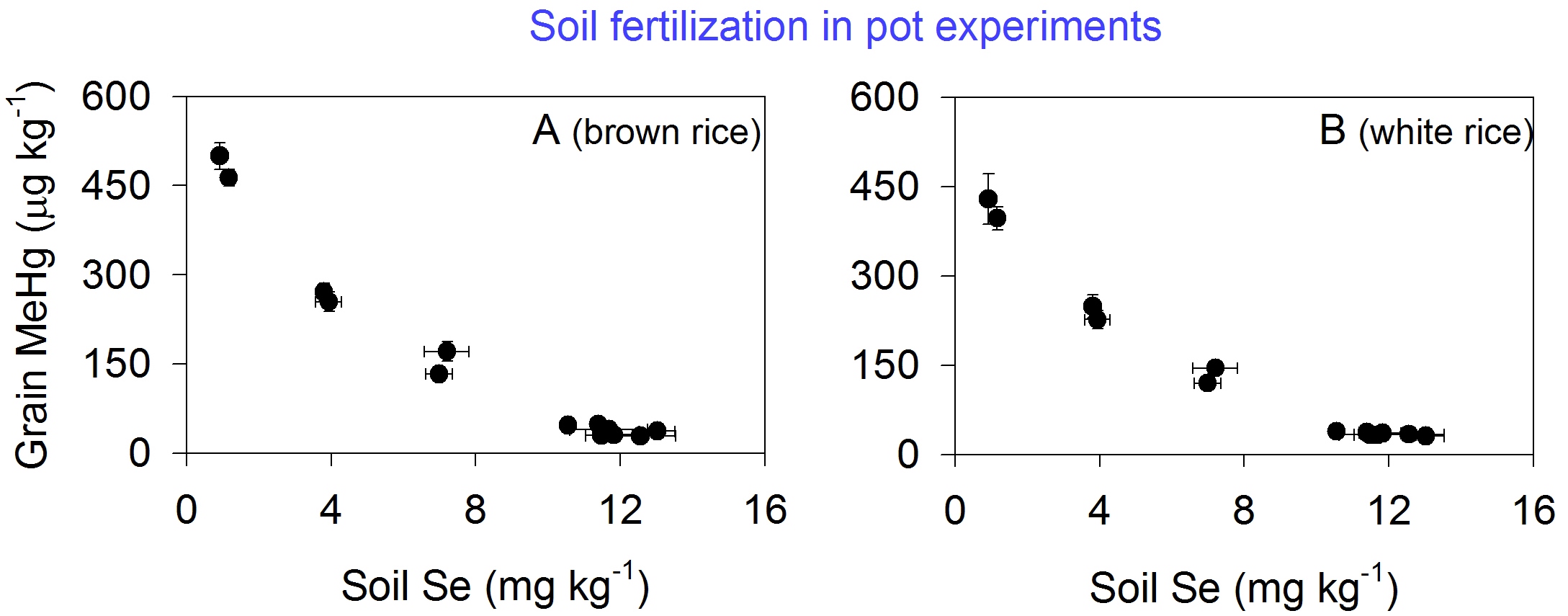
**

**Figure S5.** Distribution of MeHg and Se in different plant tissues following soil and foliar fertilization with Se in Low-Se soil. A) [MeHg] and B) [Se], soil fertilization; C) [MeHg] and D) [Se], foliar fertilization. Data for High-Se soil is not available. Data are given as means ± SD (n=3). Different letters indicate significant differences among treatments in rice plant tissues (*p* < 0.05).


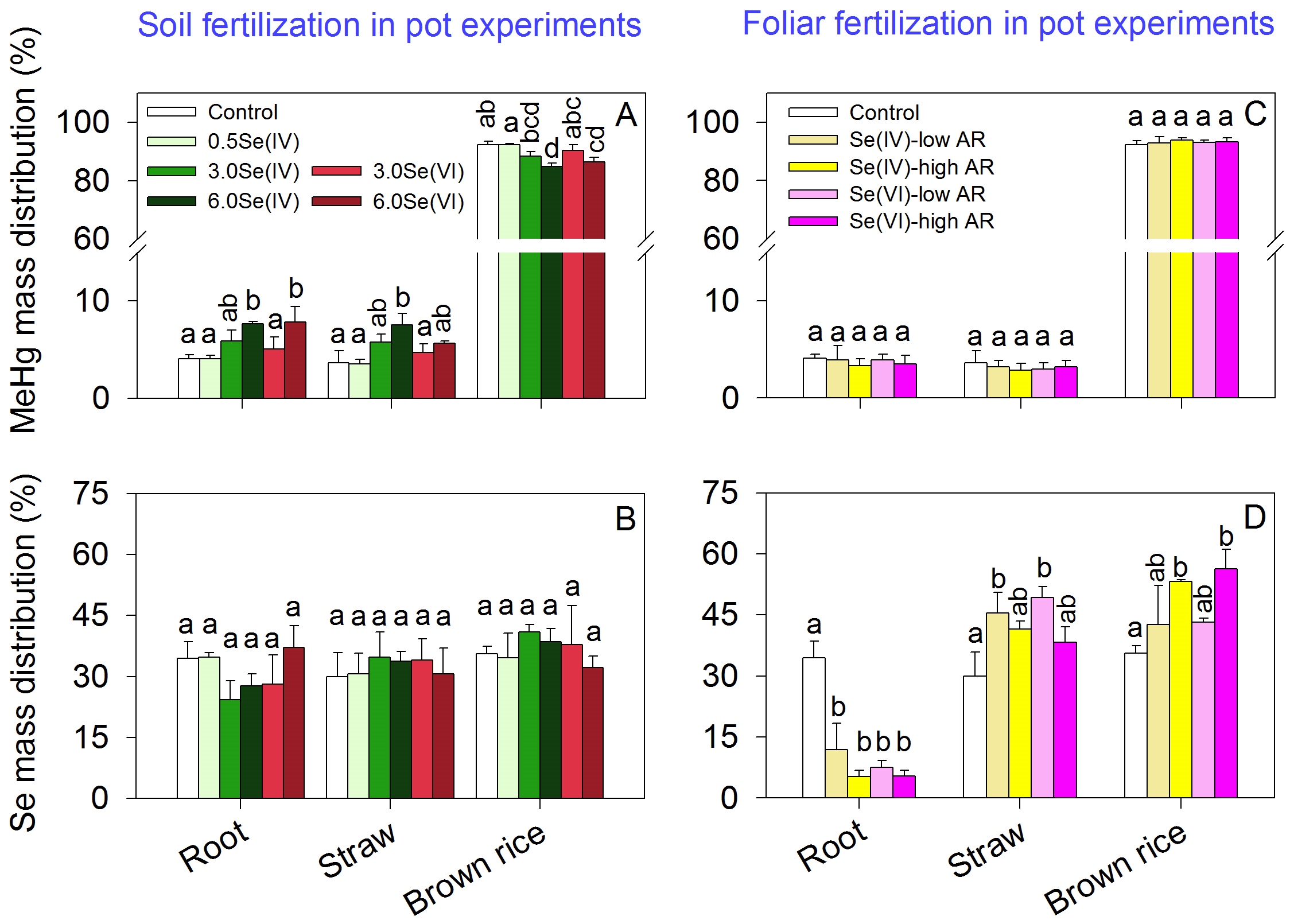


**Figure S6.** Relationships between MeHg and THg in: A) brown rice and B) white rice. Data from ‘soil fertilization’ (High-Se and Low-Se soils) and ‘foliar fertilization’ experiments are included. Each point represents one data point.


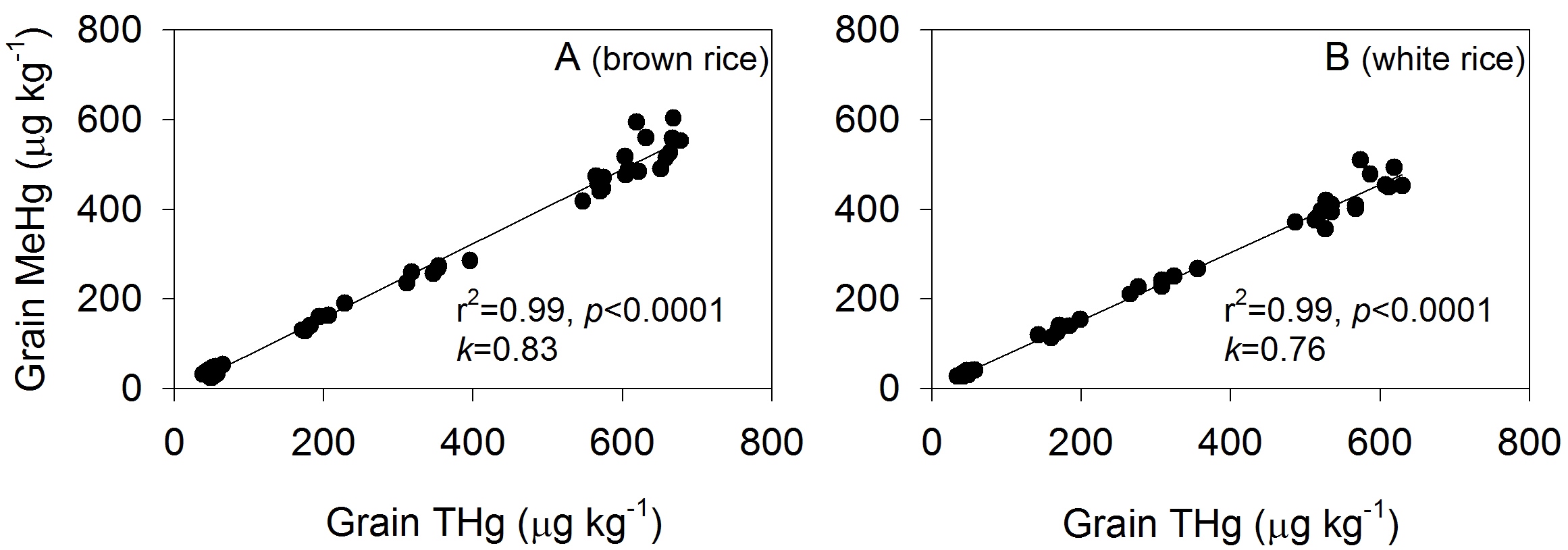


**Figure S7.** Concentrations of: A) Se and B) MeHg in soils or plant tissues following foliar fertilization with Se. Data are given as means ± SD (n = 3). Different letters indicate significant differences among treatments in soil or rice plant tissues (*p* < 0.05).

**
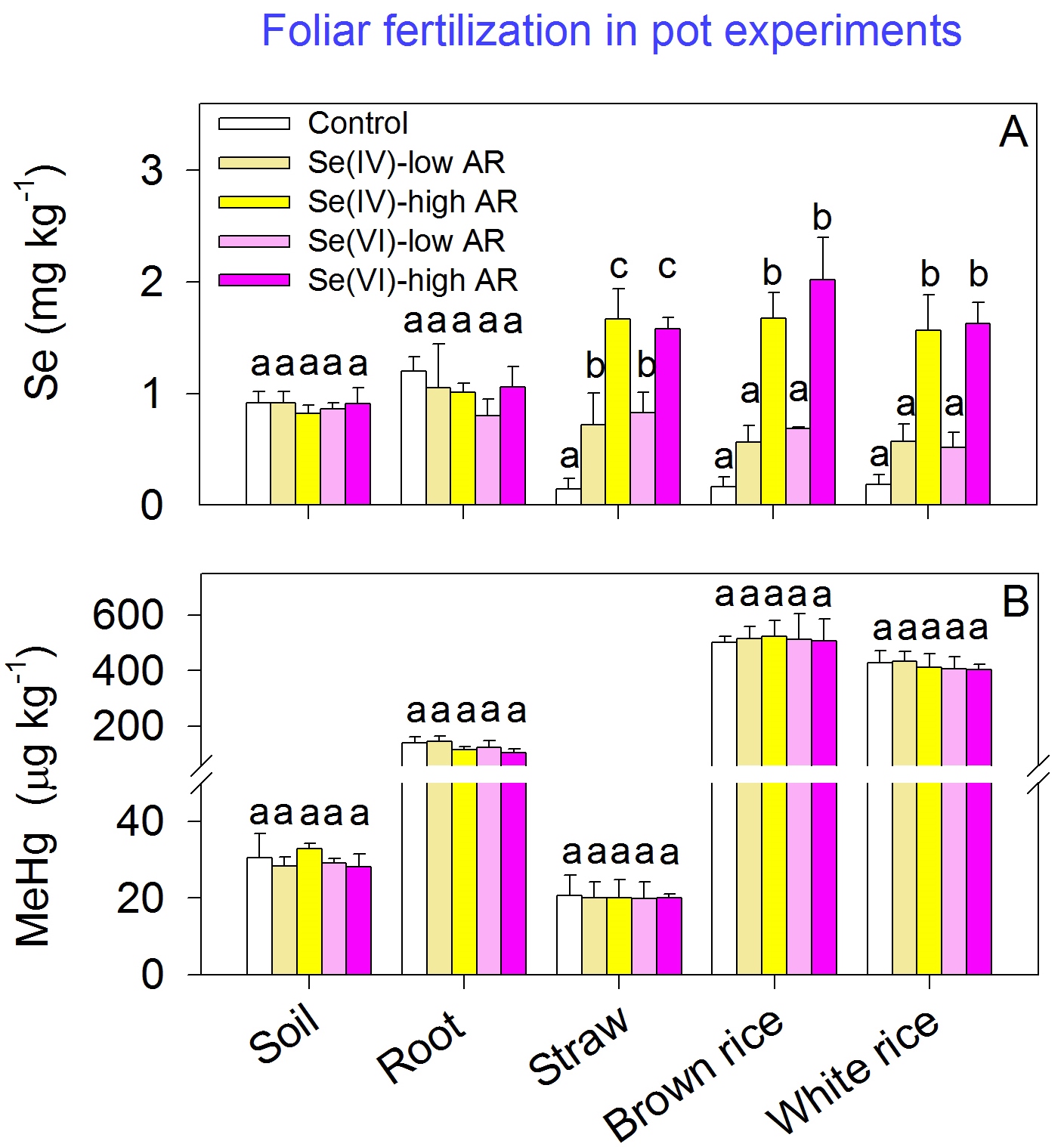
**

**Figure S8.** Total uptake of MeHg in rice plant per pot following soil fertilization (green columns) or foliar fertilization (yellow columns) with Se in Low-Se soil. Calculated as root mass×[MeHg]_root_+ straw mass×[MeHg]_straw_ + brown rice mass×[MeHg]_brown rice_. [MeHg]: MeHg concentrations in plant tissues. Data are given as means ± SD (n = 3). Different letters (lowercase letters for soil fertilization and uppercase letters for foliar fertilization) indicate significant differences among treatments (*p* < 0.05).


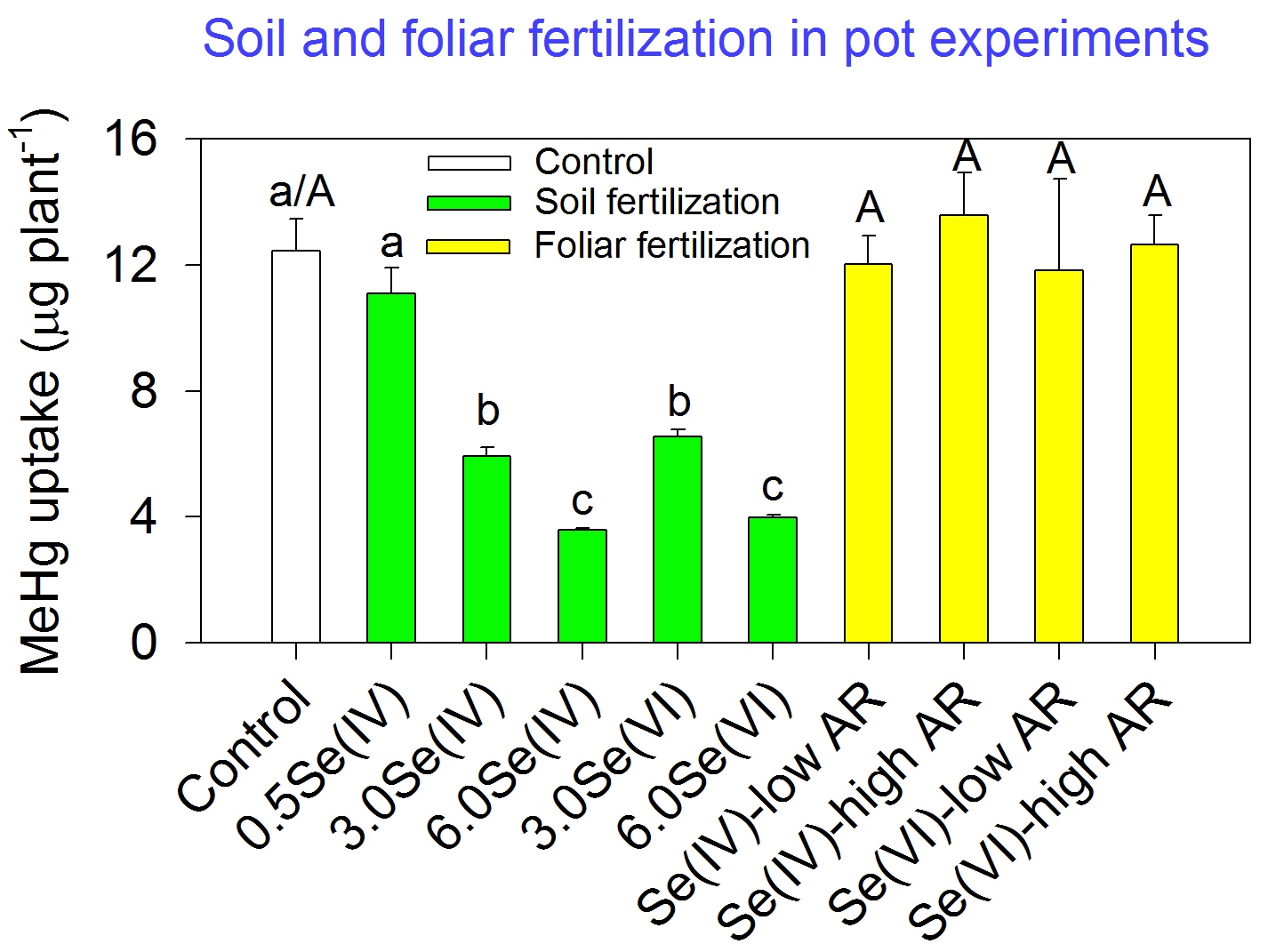


**Figure S9.** Possible mechanisms of MeHg-Se antagonism in soil-rice systems


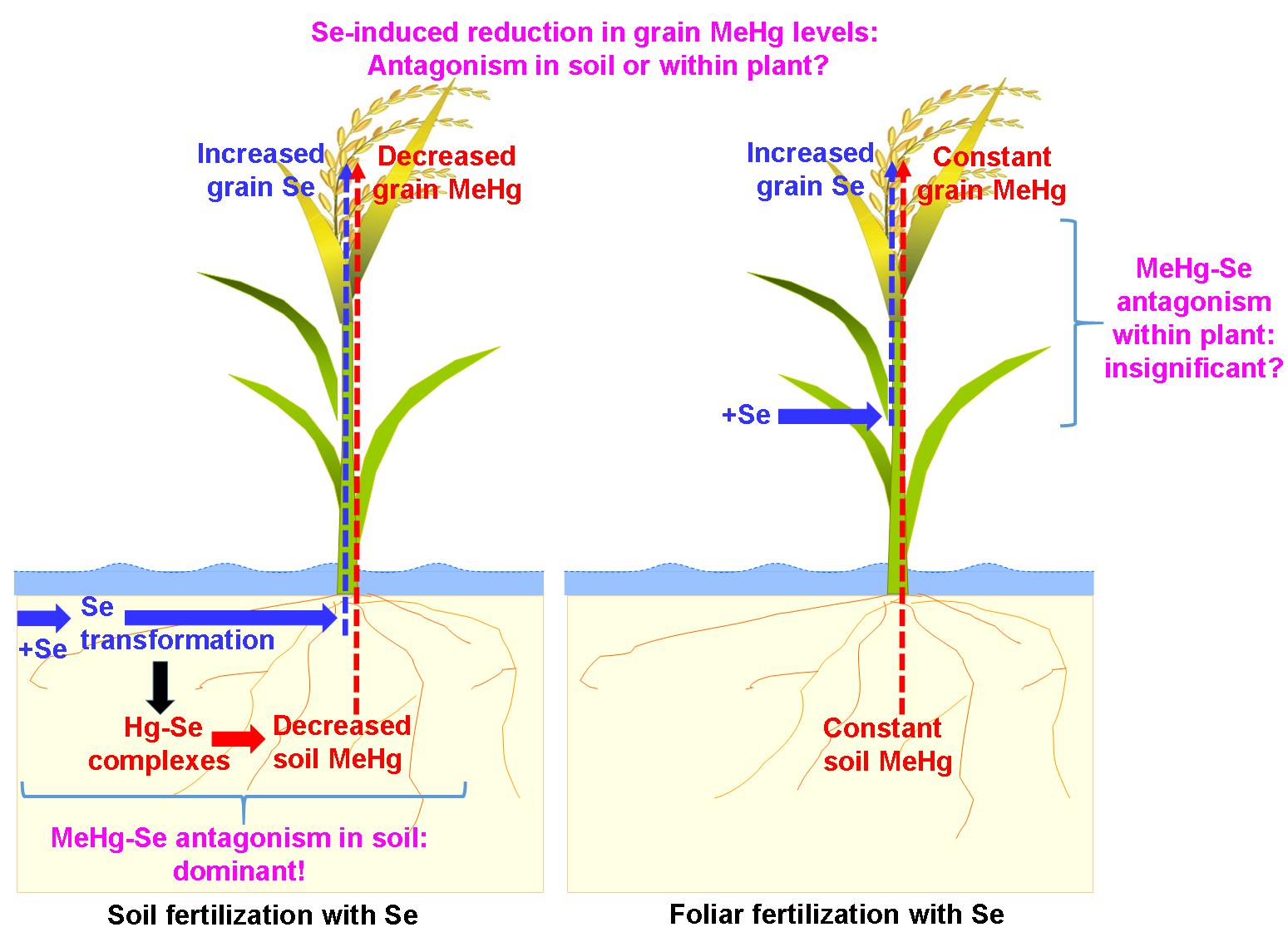


**4. References**

1. Zhong, H. & Wang, W. X. Metal-solid interactions controlling the bioavailability of mercury from sediments to clams and sipunculans. *Environ. Sci. Technol.* **40**, 3794‒3799 (2006).

2. Jonsson, S. *et al*. Differentiated availability of geochemical mercury pools controls methylmercury levels in estuarine sediment and biota. *Nat. Commun*. **5**, 4624; DOI: 10.1038/ncomms5624 (2014).

3. Ma, L., Zhong, H. & Wu, Y. G. Effects of metal-soil contact time on the extraction of mercury from soils. *Bull. Environ. Contam. Toxicol.* **94**, 399‒406 (2015).

4. Liang, L. *et al*. Re-evaluation of distillation and comparison with HNO_3_ leaching/solvent extraction for isolation of methylmercury compounds from sediment/soil samples. *Appl. Organomet. Chem.* **18**, 264−270 (2004).

5. DeWild, J. F., Olund, S. D., Olson, M. L. & Tate, M. T. Methods for the

preparation and analysis of solids and suspended solids for methylmercury. *Laboratory Analysis Section A, Water Analysis*; USGS Techniques and Methods: 5-A7, 1‒13 (2004).

6. *Method 7473*: *mercury in solids and solutions by thermal decomposition,*

*amalgamation and atomic spectrophotometry*. Washington, DC, 1998.

7. *Method 1630*: *methylmercury in water by distillation, aqueous ethylation, purge*

*and trap, and CVAFS*; EPA-821-R-01-020; Washington, DC, 2001.

8. Zhang, H. *et al*. Selenium in soil inhibits mercury uptake and translocation in rice

(*Oryza sativa* L.). *Environ. Sci. Technol.* **46**, 10040‒10046 (2012**)**.

9. Zhao, J. T. *et al*. Selenium inhibits the phytotoxicity of mercury in garlic (*Allium sativum*). *Environ. Res*. **125**, 75‒81 (2013).
